# *Margaritaria nobilis* L.F. (Phyllanthaceae): Ethnopharmacology and Application of Computational Tools in the Annotation of Bioactive Molecules

**DOI:** 10.3390/metabo12080681

**Published:** 2022-07-25

**Authors:** Johan Carlos C. Santiago, Carlos Alberto B. Albuquerque, Abraão de Jesus B. Muribeca, Paulo Roberto C. Sá, Sônia das Graças Santa R. Pamplona, Consuelo Yumiko Y. e Silva, Paula Cardoso Ribera, Enéas de Andrade Fontes-Júnior, Milton Nascimento da Silva

**Affiliations:** 1 Laboratory of Liquid Chromatography (Labcrol), Institute of Exact and Natural Sciences, Federal University of Pará, Belém 66075-110, Brazil; johan.santiago@itec.ufpa.br (J.C.C.S.); carlos.albuquerque@icen.ufpa.br (C.A.B.A.); abraao_muribeca@hotmail.com (A.d.J.B.M.); sgpamplona@yahoo.com.br (S.d.G.S.R.P.); yumikoyoshioka@yahoo.com.br (C.Y.Y.e.S.); 2 Federal Institute of Pará, Belém 68740-970, Brazil; paulo.sa@ifpa.edu.br; 3 Faculty of Pharmaceutical Sciences, Federal University of Pará, Belém 66075-110, Brazil; paularibera17@gmail.com (P.C.R.); efontes@ufpa.br (E.d.A.F.-J.)

**Keywords:** *Margaritaria nobilis*, LC-HRMS, computational tools, phenolic compounds

## Abstract

*Margaritaria nobilis* is a shrubby species widely distributed in Brazil from the Amazon to the Atlantic Rainforest. Its bark and fruit are used in the Peruvian Amazon for disinfecting abscesses and as a tonic in pregnancy, respectively, and its leaves are used to treat cancer symptoms. From analyses via UHPLC-MS/MS, we sought to determine the chemical profile of the ethanolic extract of *M. nobilis* leaves by means of putative analyses supported by computational tools and spectral libraries. Thus, it was possible to annotate 44 compounds, of which 12 are phenolic acid derivatives, 16 are *O*-glycosylated flavonoids and 16 hydrolysable tannins. Among the flavonoids, although they are known, except for kaempferol, which has already been isolated from this species, the other flavonoids (**10**, **14**, **15**, **21**, **24**–**26**, **28**–**30**, **33**–**35**, **40** and **41**) are being reported for the first time in the genus. Among the hydrolysable tannins, six ellagitannins present the HHDP group (**6**, **19**, **22**, **31**, **38** and **43**), one presents the DHHDP group (**5**), and four contain oxidatively modified congeners (**12**, **20**, **37** and **39**). Through the annotation of these compounds, we hope to contribute to the improved chemosystematics knowledge of the genus. Furthermore, supported by a metric review of the literature, we observed that many of the compounds reported here are congeners of authentically bioactive compounds. Thus, we believe that this work may help in understanding future pharmacological activities.

## 1. Introduction

The species *Margaritaria nobilis*, for a time, was classified as belonging to the genus *Phyllanthus*¸ which chemical-pharmacological knowledge is widely disseminated [1]. However, phylogenetic studies have suggested reclassification to the genus *Margaritaria*, which is currently considered [2].

This species is popularly known as “botãozinho”, “figueirinha”, “sobragirana”, “café-bravo” and “fruto-de-jacamin”, and although not endemic in Brazil, it has well-established phytogeographic domains in the Amazon, Caatinga and Atlantic Forest [3,4]. In traditional medicine, the decoction of its bark is used for asepsis of abscesses, the slightly boiled fruit is used as a pregnancy tonic [5], and the leaves are used to treat cancer-like symptoms [6].

Chemically, for the genus *Margaritaria*, the presence of phenolic derivatives, such as gallic acid and glycosylated flavonoids obtained from *M. discoidea* [7,8], and the alkaloids securinine and phyllocrisin [9], found in *M. indica*, are reported.

Beyond that, according to our literature review, there are a few phytochemical studies of *M. nobilis*, on which authors reported the presence of kaempferol, the phenols gallic acid and methyl gallate and the tannin corilagin, in the leaves of the plant; betulinic acid and the alkaloid phyllanthidine were isolated from the stem [4].

In accordance with pharmacological documents for these species, we believe that activities, such as cytotoxicity [8], antioxidant [7], anti-inflammatory [10], analgesic effect [11], antimicrobial activity [12] and leishmanicidal activity [4] can be understood in the light of the potential that these classes have.

In this regard, we opportunely emphasize that a multifaceted investigative approach to the magnitude of these activities is only possible in light of the unequivocal structural definition of these biomolecules [13]. And, in this field, although Nuclear Magnetic Resonance spectroscopy is the main technique [14], we are well supported by computational tools that, from machine training, have anticipated the structural prelude of phytoconstituents of complex matrices [15,16].

At this juncture, the workflows for mining pharmacologically relevant natural products have arguably become faster and more precise, as they provide bioguided screening and isolation of active molecules [17,18,19]. The prospect is that these advances will become increasingly significant as the sharing of scientific data becomes normatized (Aron et al. 2020). Moreover, the continuous supply of spectral data of identified compounds has served as a mirror for the prospecting of unknown compounds, disclosing new natural matrices with high therapeutic advantages [20,21].

Thus, on this and other evidence, we strongly believe that plant extracts that have never been thoroughly investigated can be satisfactorily targeted to various pharmacological segments from the chemical annotation provided by robust computational tools.

In this perspective, considering that the species *M. nobilis* possesses an authentic arsenal of chemical constituents capable of providing formidable pharmacological bioprospecting, and supported by computational tools, we sought to annotate the largest number of the compounds present in the ethanolic extract of *M. nobilis* leaves through putative analysis via UHPLC-MS/MS, followed by a metric review of the pharmacological properties of compounds already reported in the literature. Thus, we describe here the annotation of 44 compounds, of which 12 are phenolic acid derivatives, 16 are flavonoids and their *O*-glycosylated derivatives, and 16 are hydrolysable tannins.

## 2. Results

### 2.1. Characterization of Detectable Components in the EtOH Extract of Margaritaria nobilis

The characterization of detectable compounds was performed using two approaches: (1) analysis of LC-MS/MS results using cheminformatics tools, and (2) manual analysis of MS and MS/MS spectra. As a result of this process, a feature-based molecular Network (Figure S2) was generated on the GNPS platform, which allowed the annotation of *M. nobilis* metabolites.

To increase the reliability in the putative identification of the compounds, the chemotaxonomy of the Phyllanthaceae family and more precisely that of the genus *Margaritaria* was considered. As shown in Table 1, forty-four compounds (Figure S3) were identified and classified into three groups: phenolic acid derivatives, flavonoids and *O*-glycosylated derivatives and hydrolysable tannins.

#### 2.1.1. Phenolic Acids Derivatives

The main phenolic compounds identified in *M. nobilis* were found to be gallic acid (**1**), methyl gallate (**4**), ethyl gallate (**11**), *p*-coumaric acid (**9**), *O*-coumaroylgalactaric acid (**2**) and *O*-feruloylgalactaric acid (**3**). These compounds showed common losses of 44 Da (CO_2_), characteristic of this class [30]. For example, gallic acid produced [M−H]^−^ in *m*/*z* 169, fragmenting into *m*/*z* 125 [M−H−CO_2_]^−^; and methyl gallate [M−H]^−^ at *m*/*z* 183, fragmenting into *m*/*z* 168 due to loss of methyl radical [M−H−CH_3_^•^]^−•^, followed by *m*/*z* 124 due to loss of CO_2_.

The compound *O*-coumaroylgalactaric acid [M−H]^−^ at *m*/*z* 355, due to loss of coumaric acid and coumaroyl, produced the fragments at *m*/*z* 191 [M−H−C_9_H_8_O_3_]^−^ and 209 [M−H−C_9_H_6_O_2_]^−^, respectively. Similarly, the compound [M−H]^−^ at *m*/*z* 385, identified as *O*-feruloylgalactaric acid, by the loss of ferulic acid and feruloyl, produced the fragments at *m*/*z* 191 [M−H−C_10_H_10_O_4_]^−^ and 209 [M−H−C_10_H_8_O_3_]^−^, respectively.

In addition to these simple phenolic acids, ellagic acid (**18**) was identified, which presented itself as a [M−H]^−^ ion at *m*/*z* 301, and in its MS/MS spectrum it was observed loss of characteristics of 18 Da (H_2_O), 28 Da (CO) and 44 Da (CO_2_). This justify the fragments at *m*/*z* 283 [M−H−H_2_O]^−^, 229 [M−H−CO−CO_2_]^−^, 201 [M−H−2CO−CO_2_]^−^ and 185 [M−H−CO−2CO_2_]^−^ [24]. The identification of ellagic acid in the sample can be used as diagnostic for the identification of its derivatives, mainly methylated (**44**), glycosylated (**13** and **16**) and methyl-glycosylated (**27** e **36**).

Methylated ellagic acid derivatives could be identified due to loss of methyl radical (−15 Da). For example, compound [M−H]^−^ at *m*/*z* 343, identified as tri-*O*-methylellagic acid (**44**), followed by loss of •CH_3_, produced fragments at *m*/*z* 328 [M−H−CH_3_^•^]^−•^, 313 [M−H−2CH_3_^•^]^−^ and 298 [M−H−3CH_3_^•^]^−•^ [31]. *O*-glycosylated ellagic acid derivatives undergo two characteristic cleavages at the *O*-glycosidic bond: (1) a homolytic cleavage to yield a radical anion, and (2) a heterolytic cleavage to yield a negative ion. This justifies, for example, the [M−H]^−^ ion at *m*/*z* 433, identified as ellagic acid *O*-xyloside (**13**), producing the fragment *m*/*z* 300 by homolytic cleavage of the *O*-xyloside bond [M−H −C_5_H_9_O_4_^•^]^−•^, and the fragment *m*/*z* 301 by the neutral loss of the glycosidic moiety [M−H−C_5_H_8_O_4_]^−^.

Except for compounds **1** and **4**, which were previously isolated from *M. nobilis* [4], the other phenolic acid derivatives (**2**, **3**, **9**, **11**, **13**, **16**, **18**, **27**, **36** and **44**) are being reported for the first time in the genus *Margaritaria*.

#### 2.1.2. Flavonoids and *O*-Glycosylated Derivatives

Kaempferol (**42**), observed as a [M−H]^−^ ion at *m*/*z* 285, produced the fragments at *m*/*z* 255, 227 and 151, as reported in the literature [32]. These fragments were used as diagnostics for the identification of *O*-glycosylated derivatives. The compound [M−H]^−^ at *m*/*z* 447, identified as kaempferol 3-*O*-glucoside (Isomer **30** and **33**), showed fragments at *m*/*z* 285 [M−H−C_6_H_10_O_5_]^−^ and 284 [M−H−C_6_H_11_O_5_^•^]^−•^, in addition to the characteristic fragments of its aglycone. Similarly, compounds **24**, **25**, **34** and **35** presented product ions [M−H−308 Da]^−^, [M−H−294 Da]^−^, [M−H−278 Da]^−^ and [M−H−278 Da]^−^, indicating the loss of the *O*-rhamnosyl-glucoside, *O*-xylosyl-glucoside, *O*-rhamnosyl-xyloside and *O*-xyloside moiety, respectively (Figure S4).

Quercetin (**40**), observed as a [M−H]^−^ ion at *m*/*z* 301, produced the fragments at *m*/*z* 273, 257, 229, 179 and 151, as reported in the literature [32]. The compound [M−H]^−^ at *m*/*z* 463, identified as quercetin 3-*O*-glucoside (Isomer **21** and **26**), presented fragments at *m*/*z* 301 [M−H−C_6_H_10_O_5_]^−^ and 300 [M−H−C_6_H_11_O_5_^•^]^−•^, in addition to the characteristic fragments of its aglycone. Similarly, compounds **10**, **14**, **15**, **28** and **29** presented product ions [M−H−324 Da]^−^, [M−H−294 Da]^−^, [M−H−308 Da]^−^, [M−H−278 Da]^−^ and [M−H−132 Da]^−^, indicating the loss of the *O*-glucosyl-glucoside, *O*-xylosyl-glucoside, *O*-rhamnosyl-glucoside, *O*-rhamnosyl-xyloside and *O*-xyloside moiety, respectively. In addition to these, a compound [M−H]^−^ at *m*/*z* 477 was identified as an isomer of methyl quercetin 3-*O*-glucoside (**41**), differing only by the presence of a methoxyl in the B ring of quercetin, producing the fragments at *m*/*z* 315 and 314 referring to cleavages in the *O*-glycosidic bond (Figure S5).

It is noteworthy that glycosylation at the 3-*O* position of the aglycone was defined based on the intensity and ratio of the radical ion and negative ion observed in the MS/MS spectrum [33]. The presence of glycosylated flavonoids in species of the genus *Margaritaria* has already been reported in the literature [7]. However, with the exception of kaempferol, which has already been isolated from *M. nobilis* [4], the other flavonoids (**10, 14, 15, 21, 24–26, 28–30, 33–35, 40, 41**) are being reported for the first time in the genus.

#### 2.1.3. Hydrolysable Tannins: Gallotannins and Ellagitannins

For the gallotannins derivatives, compounds **8**, **23** and **32**, the number of galloyl groups can be calculated by adding *n* × C_7_H_4_O_4_ (152 Da) to the glycosidic moiety which, in this study, basically consisted of a glucose C_6_H_12_O_6_ (180 Da) and a dideoxyglucose C_6_H_12_O_4_ (148 Da). In the negative mode MS/MS spectra, gallotannins derivatives produced characteristic fragment ions, such as [M−H−152 Da]^−^ and [M−H−170 Da]^−^, denoting neutral losses of galloyl and gallic acid groups, respectively [24].

**Figure 1:**
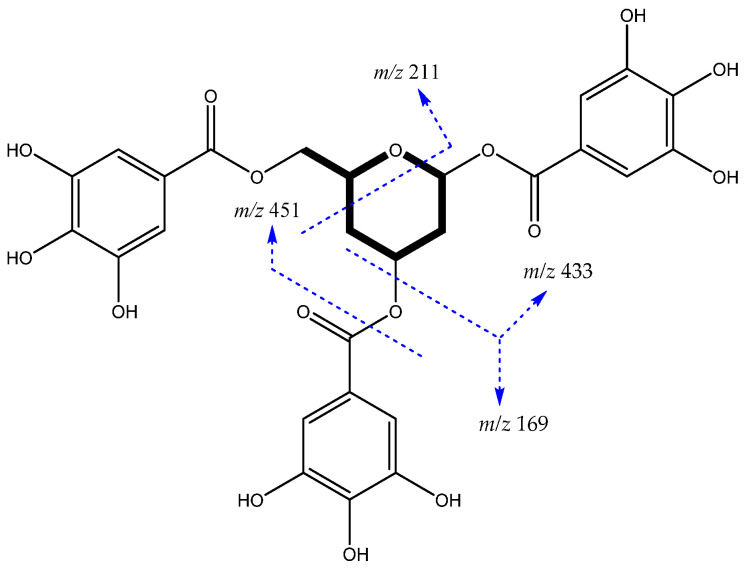
Trigalloyl-dideoxyglucose structure and main fragments.

The scheme in Figure 1 shows the main fragmentation pathways of the [M−H]^−^ ion at *m*/*z* 603, identified as trigalloyl-dideoxyglucose (**32**). In addition to the characteristic losses mentioned, the fragment ion *m*/*z* 211 probably resulted from a retro Diels–Alder mechanism (RDA) in the glycosidic portion, after the loss of gallic acid from the deprotonated molecule [M−H−gallic acid−C_11_H_10_O_5_]^−^ (see spectrum in Figure S6A).

For the identification of ellagitannins, the characteristic losses of galloyl group [M−H−152 Da]^−^, gallic acid [M−H−170 Da]^−^, HHDP group [M−H−302]^−^ and fragmentation in the DHHDP group and its oxidatively modified congeners were considered [34]. However, the differentiation between the constitutional isomers of ellagitannins is not possible to determine by mass spectrometry alone [24,34].

For this reason, the annotations were made based on the structural proposals provided by the Sirius 4 software [35], considering the systematic classification of the Canopus [36], and the proposed structural formula was chosen based on compounds of this class already reported in the genus or family of *M. nobilis*.

**Figure 2:**
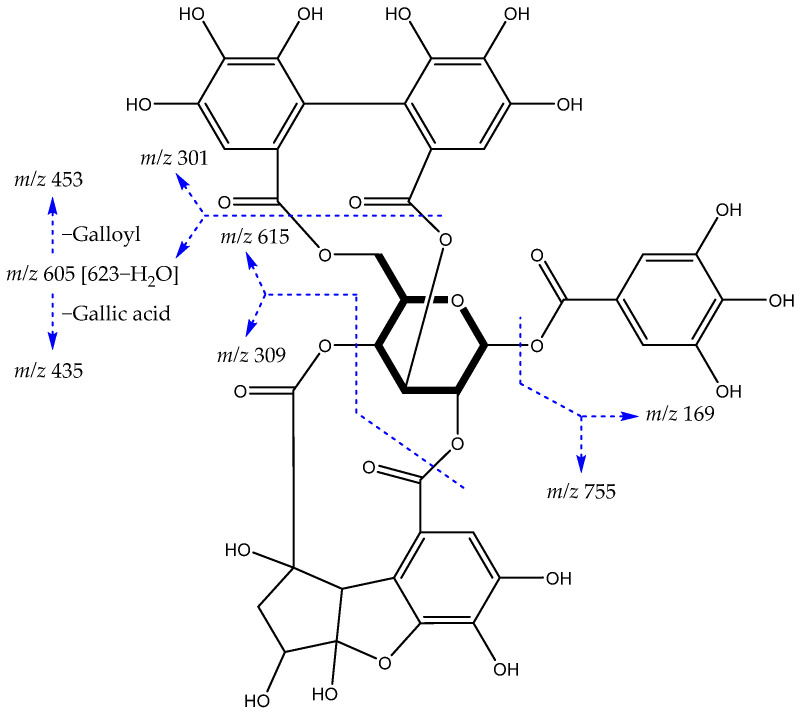
Phyllanthusiin C structure and main fragments.

In our study, six ellagitannins were putatively identified containing only HHDP groups (**6**, **19**, **22**, **31**, **38** and **43**), one containing DHHDP group (**5**), two isomers containing Che group (**7** and **17**) and four containing modified congeners oxidatively (**12**, **20**, **37** and **39**). Here, the ion [M−H]^−^ at *m*/*z* 925 taken as an example, fragmented into *m*/*z* 755 [M−gallic acid]^−^, 615 [M−H−C_13_H_10_O_9_]^−^, 605 [M−H−HHDP−H_2_O]^−^, 309 [C_13_H_10_O_9_−H]^−^ and 301 [Ellagic acid−H]^−^, which allowed its identification as an isomer of phyllanthusiin C (**12**), already isolated from the species *Phyllanthus myrtifolius* and *P. urinaria* (Phyllanthaceae) [37]. The diagram in Figure 2 presents the main fragmentation pathways of this compound.

Another four ellagitannins did not show MS/MS spectra deposited in a database or in the scientific literature, but could be annotated based on the spectral similarity observed in the molecular lattice, and evaluation of the fragmentation pattern.

**Figure 3:**
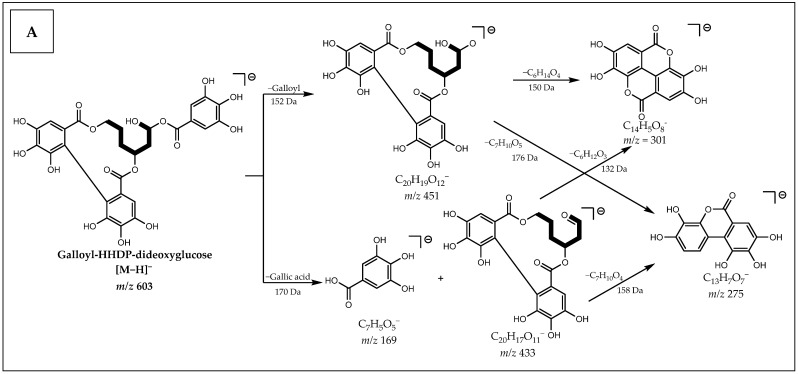

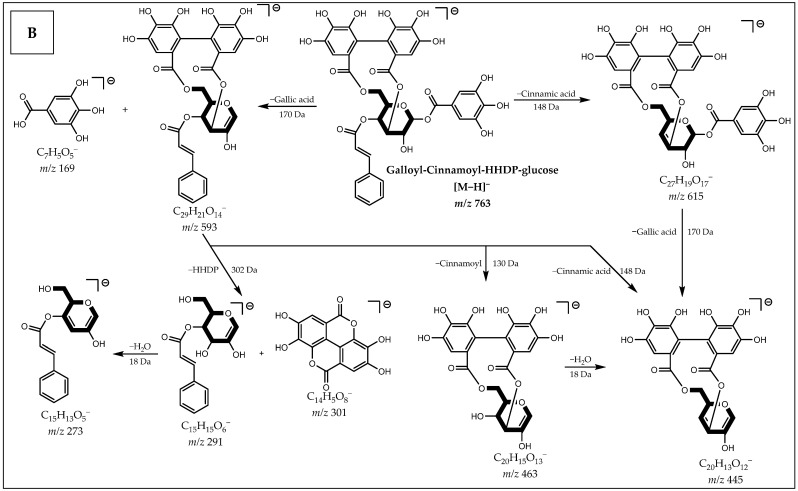
Proposal for fragmentation of: (**A**) Galloyl-HHDP-dideoxyglucose; (**B**) Galloyl-Cinnamoyl-HHDP-glucose.

For example, the [M−H]^−^ ion at *m*/*z* 603 was putatively identified as Galloyl-HHDP-dideoxyglucose (**31**) due to losses of galloyl (152 Da) and gallic acid (170 Da) forming, respectively, the fragments at *m*/*z* 451 and 433, which by splitting the HHDP group form the fragments at *m*/*z* 301 [Ellagic acid−H]^−^ and 275 [Urolithin−H]^−^, confirming the presence of a modified sugar as shown in Figure 3A (see spectrum in Figure S6B).

Analysis of the MS/MS spectrum (Figure S6C) of the [M−H]^−^ ion at *m*/*z* 763 indicates an ellagitannin of the Galloyl-Cinnamoyl-HHDP-glucose type (**43**), which is confirmed by the neutral losses of 170 Da (gallic acid), 148 Da (cinnamic acid), 130 Da (cinnamoyl) and 302 Da (Ellagic acid), in addition to neutral losses of H_2_O (18 Da) as shown in Figure 3B.

**Figure 4:**
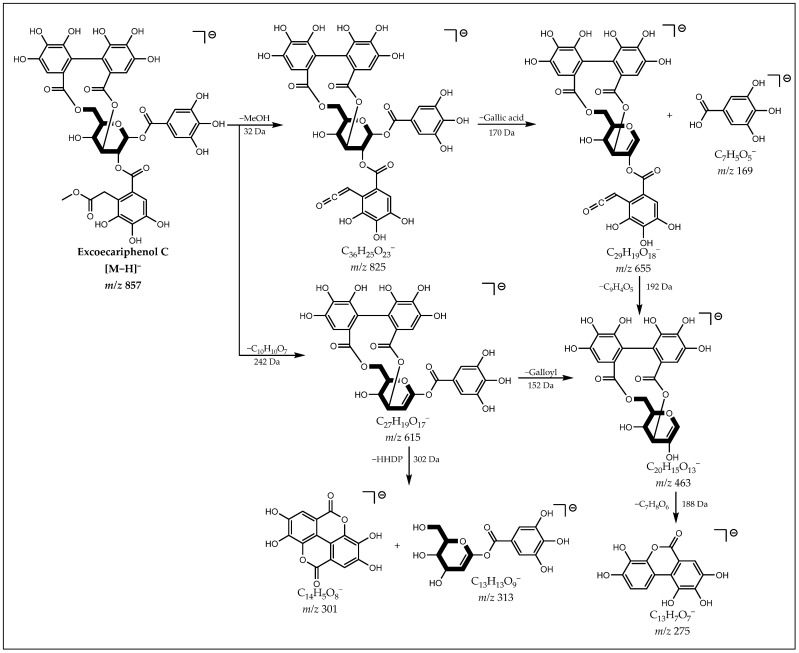
Proposal for fragmentation of Excoecariphenol C Isomer.

The ion [M−H]^−^ at *m*/*z* 857 showed the fragments at *m*/*z* 169 [Gallic acid−H]^−^, 275 [Urolithin−H]^−^ and 301 [Ellagic acid−H]^−^, indicating the presence of galloyl and HHDP groups in the structure of the deprotonated molecule, as well as the loss of 242 Da suggests a galloyl-methylacetate group. From the fragmentation proposal shown in Figure 4, it is plausible to infer that it is an ellagitannin isomer of Excoecariphenol C (**22**) (see spectrum in Figure S6D).

**Figure 5:**
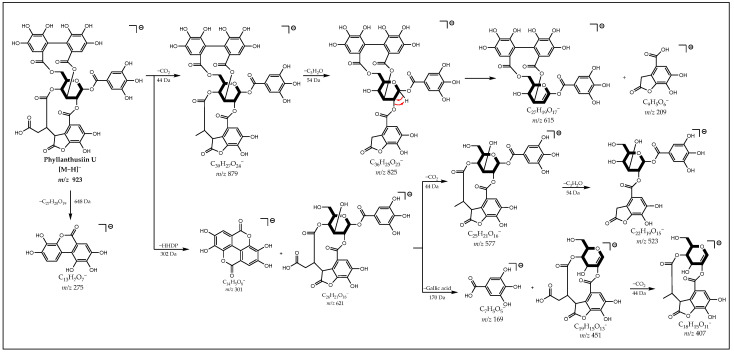
Proposal for fragmentation of Phyllanthusiin U Isomer.

The ion [M−H]^−^ at *m*/*z* 923 showed the fragments at *m*/*z* 169, 275 and 301, indicating the presence of galloyl and HHDP groups. The presence of an oxidatively modified DHHDP group can be suggested by the neutral loss of 44 Da (CO_2_) followed by 54 Da (C_3_H_2_O) from the deprotonated molecule forming, respectively, the ions *m/z* 879 and 825, which loses the residue of this group forming the ions *m*/*z* 615 and 209. Thus, from the fragmentation proposal presented in Figure 5, it is possible to suggest that it is an ellagitannin isomer of Phyllanthusiin U (**39**) (see spectrum in Figure S6E).

Research on the annotated hydrolysable tannins, carried out in a database of natural products, such as KNApSAcK and Dictionary of Natural Products, confirmed the presence of these compounds in the Phyllanthaceae family, especially in the *Phyllanthus* genus, which is closely related to *Margaritaria*. The ellagitannin corilagin has already been isolated from the species *M. nobilis* [4], and was identified in our study by mass spectrometry (compound **6**). The remaining hydrolysable tannins are being reported for the first time in the genus.

## 3. Discussion

Despite reports of the use of *Margaritaria nobilis* in traditional medicine, only one study was performed on antimycobacterial evaluation [38], as well as limited studies on the characterization of its secondary metabolites [4].

In view of this, as an alternative to the use of the barks, which compromises the integrity and perpetuation of the species, we preferred to evaluate the leaves in view of their high availability and rapid natural replacement, with the perspective that it may have interesting compounds as much as those already observed at the bark. Based on the results obtained, a search was carried out in the scientific literature on the pharmacological activities already attributed to compounds (or their class) that were putatively identified in the ethanolic extract of *M. nobilis* leaves.

As result, studies with extracts of plant species rich on glycosylated flavonoids show pharmacological activities such as analgesic and anti-inflammatory [39]. For example, rutin (**15**) produces antinociceptive effects involving central modulation of the vIPAG downstream circuit partially by an opioidergic mechanism [40]. A mixture of quercetin 3-*O*-glucoside (**21** and **26**) showed comparable antinociceptive activity to the reference compound indomethacin [41].

Kaempferol (**42**) and its glycosylated derivatives are widely distributed in nature and have several biological activities. A review of kaempferol discussed the anti-inflammatory effects and mechanisms of action of this substance, confirming its potential to improve inflammation under both in vitro and in vivo conditions [42]. Other biological effects can be attributed to these substances, such as: hepatoprotective [43], gastroprotective [44], anti-arthritis [45], anti-cancer [46] and neuroprotective [47].

Ellagic acid (**18**) is a polyphenol widely investigated for its pharmacological properties, mainly against toxicity and liver diseases, which can be justified by its antioxidant capacity, in addition to reducing the lipid profile and lipid metabolism, altering pro-inflammatory mediators and decrease factor activity (kB). In addition to being detected in its free form, ellagic acid can be released by the hydrolysis of ellagitannins under physiological conditions [48,49].

Currently, articles and patents show a growing interest in hydrolysable tannins due to their economic, chemical and biological value, which can be used as veterinary products, food additives, biopesticides and for structural bone repair. Among the biological activities, we can mention anticancer, antioxidant, antimicrobial, anti-inflammatory, antidiabetic, healing, cardiovascular protection and antiviral activity [34,50,51].

The hydrolysable tannins are subdivided into gallotannins and ellagitannins. In our analyses, three gallotannins and several ellagitannins were identified. We mention here those that were detected with the highest degree of ionization, which are the isomers of: corilagin (**6**), geraniin (**5**) and chebulagic acid (**7** and **17**).

A systematic review of the pharmacological effects of corilagin described this substance as a promising herbal agent, highlighting its good antitumor activity in hepatocellular carcinoma and ovarian cancer cells [52]. Recently, this substance was tested as a non-nucleoside inhibitor of SARS-CoV-2, the virus that causes COVID-19. The results of this study indicate that this substance has great potential to become a new and effective drug to treat patients infected with this virus [53].

Geraniin has also been shown to be a promising therapeutic agent against SARS-CoV-2, inhibiting the entry of the virus into human cells [54]. Another study reports the potential of this substance against hepatitis B virus (HBV), interfering with the synthesis, stability or transcription of viral DNA [55]. A comprehensive review of this substance found its diversity of bioactive properties, with recommendations for additional studies for possible applications in the food, cosmetic and pharmaceutical industries [56].

The promising pharmacological potential of ellagitannins is undeniable, and we cite as a last example chebulagic acid, which was evaluated for its inhibition of the pleiotropic cytokine TNFα that induces pro-inflammatory and pro-angiogenic changes, configuring this compound as an anti-inflammatory agent [57]. Another test performed with this compound showed antiviral activity, which may represent a potential therapeutic agent to control enterovirus 71 infections [58].

## 4. Materials and Methods

### 4.1. Chemicals and Reagents

Sodium hypochlorite P.A. was acquired from Dinâmica (Jaraguá do Sul, SC, Brazil). Ethyl alcohol (99%) was purchased from Êxodo Científico (Sumaré, SP, Brazil). Acetonitrile Grade LC-MS and formic acid were purchased from Merck (Darmstadt, Germany). Ultrapure water was obtained by a Direct-Q 5 system (Millipore, Merck Darmstadt, Germany).

### 4.2. Botanical Collection and Identification

Approximately 1 (one) kilogram of green and homogeneous leaves of mature specimens of *Margaritaria nobilis* were collected in the forest region of the municipality of Bragança/PA, Brazil, under the coordinates (1°02′08″ S and 46°49′41″ W). The botanical identification was carried out at the Embrapa Amazônia Oriental institution, by the botanist Nascimento, E.A.P., with an exsiccata deposited in the IAN herbarium, in the same institution, under registration number 191496. After the botanical certification, the material was washed with 0.1% sodium hypochlorite solution (NaCℓO) to eliminate micro-organisms (fungi, bacteria, etc.), then with distilled water to remove residues and sprinkled with absolute ethanol for asepsis. Then, the material was dried in a circulation oven (Quimis, Diadema, Brazil) at 45 °C until constant weight.

### 4.3. Obtaining the Ethanol Extract

The dried leaves were ground in a ball mill (Fritsch, Idar-Oberstein, Germany) until obtaining a semi-fine powder granulometry (60–100 µm). The crushed material was subjected to a 48-h extraction divided into two 24-h batches, using ethanol (99%) as solvent in the proportion of 4 L of solvent for each 1.0 kg of dry and crushed material. Subsequently, the volumes were pooled and concentrated in a rotary evaporator (Büchi, Flawil, Germany). The concentrate was oven dried at 40 °C to constant weight.

### 4.4. Sample Preparation for Analysis via UHPLC-MS/MS

The extract (10 mg) was subjected to a pre-treatment by solid phase extraction (SPE) in a H_2_O:MeOH 2:8 (*v*/*v*) system to retain interferences, especially fat and chlorophyll present in the leaves. For this, a C18 analytical cartridge (SPE, Phenomenex, Torrance, CA, USA) was used with 50 mg of stationary phase and a volume of 1 mL, previously conditioned with 1 mL of MeOH and 1 mL of ultrapure water. After SPE treatment, a 3-mg aliquot was solubilized in 1 mL of a 2:8 H_2_O:MeOH system, followed by filtration with a 0.22 µm hydrophilic syringe filter (Millipore, Merk, Darmstadt, Germany) for analysis.

### 4.5. Analysis via UHPLC-ESI-QToF-MS/MS

The matrix was analyzed in an ultra-performance liquid chromatography system coupled to an ESI-QToF Xevo G2-S mass spectrometer (Waters Corp., Mil-ford, MA, USA) with an electrospray ionization (ESI) source operating in negative ionization mode. The mass scan had a range of 100 to 1200 Da and leucyin-enkephalin was used as the Lockspray reference mass.

UHPLC analysis was performed on a BEH C18 column (50 × 2.1 mm, 1.7 µm) Waters. The column and autoinjector temperatures were maintained at 40 and 25 °C, respectively. The chromatography run was performed with ultrapure water (solvent A) and acetonitrile (solvent B), both acidified with 0.1% formic acid. The gradient method was defined as follows: 0 min—10% B; 2 min—20% B; 30 min—50% B. The flow rate was 300 µL/min, and the injection volume was 2.00 µL. The total ion chromatogram was acquired using Masslynx V4.1 software (Waters Corp., Milford, MA, USA). The mass spectrometry parameters were set to the following: desolvation gas flow (N_2_) at 800 L/h and desolvation temperature at 450 °C, cone gas flow (N_2_) at 50 L/h, source temperature at 120 °C. The capillary and sampling cone voltages were set to 2.0 kV and 80 V, respectively.

Data-dependent acquisition (DDA, MS/MS) was performed on the five most abundant ions detected in full-scan MS (top 5 experiments per scan). The ion peaks were detected at charge states +1 and +2 with the inclusion of the 10 most intense ion peaks with a charge state tolerance of 0.2 Da (*m*/*z*) and an extraction tolerance of 2 Da. The differentiation of molecular ions, adducts and fragment ions were performed by chromatographic deconvolution with 3 Da isotope tolerance and 6 Da isotope extraction tolerance. The MS/MS isolation window width was 1 Da, and the scaled normalized collision energy (NCE) was set to units of 10, 20, 30, 40 and 50 eV.

### 4.6. Processing of UHPLC-MS/MS Data

UHPLC-MS/MS data were converted from standard .raw format (Waters Corp., Milford, MA, USA) to .mzML format using MSConvert 3.0.2 software [59]. The resulting file was processed using MZmine v2.53 [60]. For mass detection, at MS^1^ and MS^2^ levels, cut-off levels of 5.0 × 10^3^ and 1.0 × 10^3^, respectively, were used. The ADAP chromatogram creation algorithm was used and set to a minimum scan group size of 3, minimum group intensity threshold of 5.0 × 10^3^, and highest maximum intensity of 5.0 × 10^3^ with an *m*/*z* tolerance of 0.002 Da. The ADAP algorithm (Wavelets) was used for the deconvolution of the chromatogram. The S/N intensity window was used as the S/N estimator with a signal-to-noise ratio set to 15, a minimum feature height of 5.0 × 10^3^, a coefficient area limit of 50, a peak duration ranging from 0.01 to 1.0 min and an RT wavelet range of 0.01 to 0.1 min, an *m*/*z* interval for MS^2^ scan pairing of 0.02 Da and an R/T interval for MS^2^ scan pairing of 0.2 min were also used. Isotopes were detected using the isotope peak grouper with an *m*/*z* tolerance of 0.02 Da, an RT tolerance of 0.2 min (absolute) and the maximum load set to 2 and the representative isotope used was the most intense. Finally, using the peak list lines filter option, features without an associated MS^2^ spectrum were removed, also using the parameter consecutive minimum peaks as 1 and minimum peaks in an isotope pattern as 1 as well. Finally, a manual validation step was performed to exclude false features, such as fragments from the ionization source [61] and features with low quality MS^2^ spectra, resulting in a final list containing 151 features.

### 4.7. Resource-Based Molecular Network Creation

From the .mgf and .csv files obtained from processing the raw data with MZmine 2.53, a Molecular Network was created using the Feature-Based Molecular Networking workflow [62] on the GNPS platform (https://gnps.ucsd.edu/ProteoSAFe/static/gnps-splash.jsp) (accessed on 1 April 2022). The precursor ion mass and MS/MS fragment ion tolerances were both set at 0.02 Da. A molecular network was then created in which the edges were filtered to have a cosine score above 0.65 and more than 4 corresponding peaks. The edges between two nodes were kept in the network only if each of the nodes appeared in each of the other 10 most similar top nodes. The molecular family size was set to a maximum of 100, and the lowest scoring borders were removed from the molecular families until the molecular family size was below this threshold. The spectra on the network were searched against the GNPS spectral libraries [63]. The library spectra were filtered in the same way as the input data. All games held between the network spectra and the library spectra were required to have a score above 0.65 and at least 4 peaks combined. Molecular networks were visualized using Cytoscape software version 3.8.0 [64]. Molecular networking work can be publicly accessed at https://gnps.ucsd.edu/ProteoSAFe/status.jsp?task=72419d61c18f424d9544b41bc32c87e9 (accessed on 7 April 2022). 

### 4.8. Putative Identification of Compounds

An extensive search in the scientific literature was carried out in order to build an internal database for the genus *Margaritaria* (Table S2), which resulted in 28 compounds already isolated from species of the genus. This table was used to evaluate the chemo-taxonomy of the *M. nobilis* species and, adjunct to the molecular network created, served as a guide for the putative identification of the compounds present in the matrix under study. MS/MS spectra that did not have any correspondence on the GNPS platform were annotated using Sirius 4 software, in addition to being compared with spectral data present in the scientific literature.

## 5. Conclusions

From a workflow based on previous chemical reports from species of the genus *Margaritaria*, as well as supported by high-performance computational tools, we were able to establish a chemical profile for the ethanolic extract of *M. nobilis* leaves. In our results, 44 compounds were annotated; among these, we highlight compounds ellagic acid, galloyl-HHDP-glucose, quercetin 3-*O*-glucoside and galloyl-Che-HHDP-glucose that, in the first instance, may support the understanding of expected pharmacological activities for the species. We also highlight that by UHPLC-MS, we were able to analyze trace compounds that in conventional methods would not be verified. We emphasize that monitoring the availability of these compounds is also important, since the magnitude of the bioactive profile of this species can change dramatically due to seasonality.

Finally, we understand that, through this work, we contributed to the knowledge of the chemical profile of the leaves of this species, providing valuable information for the understanding and certification of pharmacological activities that will be studied in the future.

## Figures and Tables

**Table 1:** Characterization of chemical compound of the extract from the leaves of *M. nobilis* by UHPLC-MS/MS in negative mode.

Peak	R.T. (min)	[M−H]^−^ Exp. (Error, ppm)	Molecular Formula	Characteristic Ions (MS^2^)	Putative Identification	Spectrum Reference
1	1.84	169.0138 (0.6)	C_7_H_6_O_5_	**125**	Gallic acid	^a^ CCMSLIB00004691622
2	4.51	355.0661 (1.1)	C_15_H_16_O_10_	337, 313, 209, **191**, 163, 147, 129	*O*-Coumaroylgalactaric acid	^a^ CCMSLIB00005745086
3	4.91	385.0766 (1.3)	C_16_H_18_O_11_	209, **191**, 173, 147	*O*-Feruloylgalactaric acid	[22]
4	5.17	183.0285 (4.4)	C_8_H_8_O_5_	**168**, 124	Methyl gallate	[23]
5	5.92	951.0703 (3.9)	C_41_H_28_O_27_	933, 915, 763, 633, 463, 461, 443, **301**, 275, 273, 169	Galloyl-DHHDP-HHDP-glucose	[24]
6	6.09	633.0710 (2.8)	C_27_H_22_O_18_	463, **301**, 275, 249, 169	Galloyl-HHDP-glucose	^a^ CCMSLIB00000847042
7	6.56	953.0888 (0.8)	C_41_H_30_O_27_	935, 909, 801, 783, 765, 633, 481, 463, 337, 319, **301**, 293, 275, 249, 169	Galloyl-Che-HHDP-glucose Isomer I	^a^ CCMSLIB00004692930
8	6.56	635.0866 (2.8)	C_27_H_24_O_18_	465, 313, 271, 221, 211, 193, **169**, 125	Trigalloyl-glucose	^a^ CCMSLIB00000845184
9	6.92	163.0389 (3.7)	C_9_H_8_O_3_	**119**	*p*-Coumaric acid	^a^ CCMSLIB00005741418
10	6.98	625.1368 (5.9)	C_27_H_30_O_17_	301, **300**, 271, 255, 243, 179, 151	Quercetin 3-*O*-glucosyl-glucoside	^a^ CCMSLIB00000847258
11	7.18	197.0445 (2.5)	C_9_H_10_O_5_	**169**, 168, 125, 124	Ethyl gallate	^a^ CCMSLIB00006691851
12	7.24	925.0983 (3.6)	C_40_H_30_O_26_	755, 615, 605, 453, 435, 309, **301**, 275, 249, 247, 169	Phyllanthusiin C Isomer	[25]
13	7.55	433.0410 (0.7)	C_19_H_14_O_12_	**301**, 300	Ellagic acid *O*-xyloside	[26]
14	7.67	595.1321 (3.7)	C_26_H_28_O_16_	301, **300**, 271, 255, 243, 179, 151	Quercetin 3-*O*-xylosyl-glucoside	^a^ CCMSLIB00004718534
15	7.84	609.1427 (4.8)	C_27_H_30_O_16_	301, **300**, 271, 255, 243, 179, 151	Quercetin 3-*O*-rhamnosyl-glucoside	^a^ CCMSLIB00005778075
16	7.87	447.0585 (4.7)	C_20_H_16_O_12_	301, **300**	Ellagic acid *O*-rhamnoside	[27]
17	7.96	953.0904 (0.8)	C_41_H_30_O_27_	935, 909, 801, 783, 765, 633, 481, 463, 337, 319, **301**, 293, 275, 249, 169	Galloyl-Che-HHDP-glucose Isomer II	^a^ CCMSLIB00004692930
18	8.01	300.9972 (4.0)	C_14_H_6_O_8_	**283**, 245, 229, 201, 185, 173, 145	Ellagic acid	^a^ CCMSLIB00004694147
19	8.39	785.0847 (1.3)	C_34_H_26_O_22_	**633**, 615, 463, 301, 275, 249, 169	Digalloyl-HHDP-glucose	[23]
20	8.39	985.1155 (0.3)	C_42_H_34_O_28_	783, 633, 463, 351, **301**, 169	Methyl neochebulagate Isomer	[23]
21	8.62	463.0890 (2.8)	C_21_H_20_O_12_	301, **300**, 271, 255, 243, 179, 151	Quercetin 3-*O*-glucoside Isomer I	^a^ CCMSLIB00004684243
22	8.73	857.1077 (3.3)	C_37_H_30_O_24_	825, 655, 615, 463, **301**, 275, 169	Excoecariphenol C Isomer	N/A
23	8.73	787.0977 (2.2)	C_34_H_28_O_22_	635, 617, 593, 465, 449, **169**	Tetragalloyl-glucose	^a^ CCMSLIB00004719474
24	8.76	593.1528 (3.7)	C_27_H_30_O_15_	285, **284**, 255, 227, 151	Kaempferol 3-*O*-rhamnosyl-glucoside	^a^ CCMSLIB00005743498
25	8.87	579.1376 (4.0)	C_26_H_28_O_15_	285, **284**, 255, 227, 151	Kaempferol 3-*O*-xylosyl-glucoside	^a^ CCMSLIB00004706607
26	8.87	463.0898 (4.5)	C_21_H_20_O_12_	301, **300**, 271, 255, 243, 179, 151	Quercetin 3-*O*-glucoside Isomer II	^a^ CCMSLIB00004684243
27	8.93	491.0852 (5.3)	C_22_H_20_O_13_	**313**, 298, 285, 270	Di-*O*-Methyl ellagic acid *O*-glucoside	^a^ CCMSLIB00004715986
28	9.41	579.1350 (0.0)	C_26_H_28_O_15_	301, **300**, 271, 255, 243, 179, 151	Quercetin 3-*O*-rhamnosyl-xyloside	^a^ CCMSLIB00004678837
29	9.61	433.0765 (1.4)	C_20_H_18_O_11_	**300**, 301, 271, 255, 243, 179, 151	Quercetin 3-*O*-xyloside	^a^ CCMSLIB00004718550
30	9.70	447.0935 (1.8)	C_21_H_20_O_11_	285, **284**, 255, 227, 151	Kaempferol 3-*O*-glucoside Isomer I	^a^ CCMSLIB00004683728
31	9.95	603.0945 (6.8)	C_27_H_24_O_16_	451, 433, 301, 275, **169**	Galloyl-HHDP-dideoxyglucose	N/A
32	10.15	603.1013 (4.5)	C_27_H_24_O_16_	451, 433, 211, **169**	Trigalloyl-dideoxyglucose	N/A
33	10.24	447.0914 (2.9)	C_21_H_20_O_11_	285, **284**, 255, 227, 151	Kaempferol 3-*O*-glucoside Isomer II	^a^ CCMSLIB00004683728
34	10.61	563.1431 (5.3)	C_26_H_27_O_14_	285, **284**, 255, 227, 151	Kaempferol 3-*O*-rhamnosyl-xyloside	[28]
35	10.69	417.0836 (3.4)	C_20_H_18_O_10_	285, **284**, 255, 227, 151	Kaempferol 3-*O*-xyloside	^a^ CCMSLIB00005739911
36	10.78	461.0736 (3.5)	C_21_H_18_O_12_	315, **300**	Methylellagic acid *O*-rhamnoside	[26]
37	11.01	951.0743 (0.3)	C_41_H_28_O_27_	907, 781, 737, 649, 615, 605, 497, 479, 435, 335, **301**, 291, 275, 273, 247, 169	Phyllanthusiin A Isomer	[25]
38	12.10	937.0962 (1.6)	C_41_H_30_O_26_	785, 767, 635, 615, 465, **301**, 275, 249, 169	Trigalloyl-HHDP-glucose	[29]
39	12.29	923.0801 (1.1)	C_40_H_28_O_26_	879, 825, 655, 621, 615, 577, 523, 451, 407, **301**, 275, 249, 169	Phyllanthusiin U Isomer	N/A
40	14.00	301.0334 (4.7)	C_15_H_10_O_7_	273, 257, 229,179, **151**, 121, 107	Quercetin	^a^ CCMSLIB00004691125
41	14.91	477.1018 (3.1)	C_22_H_22_O_12_	**314**, 315	Methylquercetin 3-*O*-glucoside	^a^ CCMSLIB00004678842
42	16.91	285.0399 (0.0)	C_15_H_10_O_6_	267, 255, 243, 239, 229, 227, 185, 163, **151**	Kaempferol	^a^ CCMSLIB00004691748
43	18.14	763.1154 (0.9)	C_36_H_28_O_19_	615, 593, 463, 445, **301**, 275, 249, 169	Galloyl-Cinnamoyl-HHDP-glucose	N/A
44	19.04	343.0450 (1.2)	C_17_H_12_O_8_	328, 313, **298**, 285, 270, 257, 242	Tri-*O*-methylellagic acid	[27]

Note: ^a^ Annotation referenced in the GNPS library; N/A—not available, annotation was made by correspondence in silico; HHDP—hexahydroxydiphenoyl; DHHDP—dehydrohexahydroxydiphenoyl; Che—chebuloyl; R.T.—retention time; Exp.—experimental. Most intense fragment in bold.

## Data Availability

The data presented in this study are available in the main article and the supplementary materials.

## Supplementary Materials

The following supporting information can be downloaded at: https://www.mdpi.com/article/10.3390/metabo12080681/s1. Figure S1: LC-MS Base Peak Intensity (BPI) chromatogram of the EtOH extract from *Margaritaria nobilis* leaves (negative mode). The selected chromatographic peaks are annotated with peak numbers referred to in Table 1; Table S1. Summary of compound-dependent parameters used in the UHPLC-ESI-QToF-MS/MS experiment; Figure S2. Molecular network from UHPLC-MS/MS data in the negative ion mode for *Margaritaria nobilis* leaf extract; Figure S3. Proposed structures for annotated metabolites in the ethanolic extract of *Margaritaria nobilis* leaves; Figure S4. General fragmentation scheme and MS/MS spectra of *O*-glycosylated kaempferol derivatives; Figure S5. *O*-glycosylated quercetin derivatives MS/MS spectra; Figure S6. MS/MS spectra of hydrolysable tannins annotated in silico; Table S2. In-house database of compounds reported in the genus *Margaritaria* (Phyllanthaceae).
