# Supplementary material for: Margaritaria nobilis L.F. (Phyllanthaceae): Ethnopharmacology and Application of Computational Tools in the Annotation of Bioactive Molecules
Source: Metabolites. 2022 Jul 25;12(8):681. doi: 10.3390/metabo12080681 (PMC9330776; doi:10.3390/metabo12080681)
Supplement: Supplementary file 1 [file metabolites-12-00681-s001.zip › metabolites-1793099-supplementary.pdf]

metabolites

MDPI

Supplementary Material

for

Margaritaria nobilis L.f. (Phyllanthaceae): Ethnopharmacology and Application of Computational Tools in the Annotation of Bioactive Molecules

Johan Carlos C. Santiago [1], Carlos Alberto B. Albuquerque [1], Abraão de Jesus B. Muribeca [1], Paulo Roberto C. Sá [2], Sônia das Graças Santa R. Pamplona [1], Consuelo Yumiko Y. e Silva [1,3], Paula Cardoso Ribera [3], Enéas de Andrade Fontes-Júnior [3] and Milton Nascimento da Silva [1,*]

[1] Laboratory of Liquid Chromatography (Labcrol), Institute of Exact and Natural Sciences, Federal University of Pará, 66075-110, Brazil; johan.santiago@itec.ufpa.br (J.C.C.S.); carlos.albuquerque@icen.ufpa.br (C.A.B.A.); abraao_muribeca@hotmail.com (A.d.J.B.M.); sgpamplona@yahoo.com.br (S.d.G.S.R.P.); yumikoyoshioka@yahoo.com.br (C.Y.Y.e.S.);
[2] Federal Institute of Pará, 68740-970, Brazil; paulo.sa@ifpa.edu.br (P.R.C.S.);
[3] Faculty of Pharmaceutical Sciences, Federal University of Pará, 66075-110, Brazil; pauIaribera17@gmail.com (P.C.R.); efontes@ufpa.br (E.d.A.F.J.).
* Correspondence: yumilton@yahoo.com.br (M.S); Tel.: +55-91-3201-7365

Figure S1. LC-MS Base Peak Intensity (BPI) chromatogram of the EtOH extract from *Margaritaria nobilis* leaves (negative mode). The selected chromatographic peaks are annotated with peak numbers referred to in Table 1.

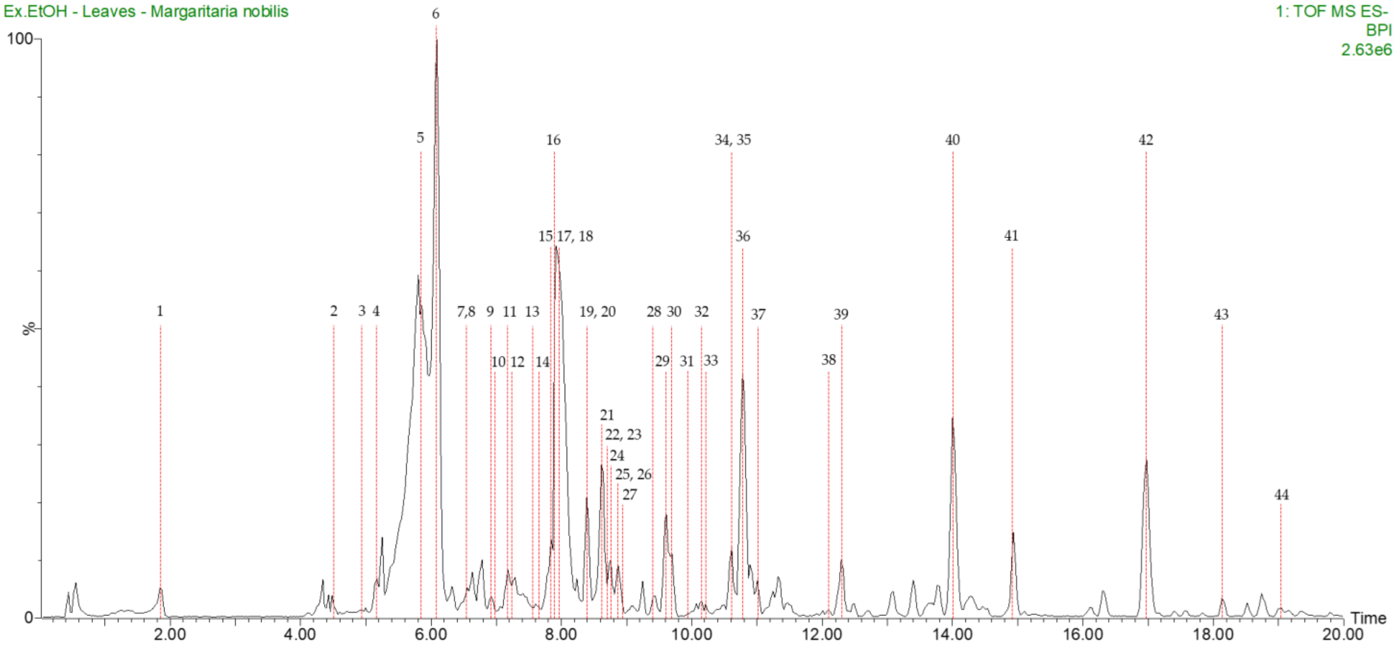

Table S1. Summary of compound-dependent parameters used in the UHPLC-ESI-QToF-MS/MS experiment.

LC parameters	Value
Column	BEH C18 Waters (50 × 2.1 mm, 1.7 µm)
Column temperature	40 °C
Autosampler temperature	25 °C
Mobile phase (A)	Ultra-pure water acidified with 0.1% formic acid
Mobile phase (B)	Acetonitrile acidified with 0.1% formic acid
Injection volume	2 µL
Flow rate	300 µL/min
Gradient method	0 min - 10% B; 2 min - 20% B; 30 min - 50% B.

Ion Source parameters	ESI(−) mode
Acquisition mode	Resolution
Capillary voltage	2.0 kV
Cone voltage	40 V
Source temperature	120 °C
Desolvation gas temperature	450 °C
Desolvation gas flow	800 L/h
Cone gas flow	50 L/h
Lockspray capillary voltage	2.5 kV

Mass Spectrometry acquisition parameters	Value
MS Survey Da Range	*m/z* 100-1200
Intensity threshold	1E2
Data format	Centroid
Fragmented ion for each scan	5
Scan rate	5 s
Charge states	−1 and −2
Collision Energy Profile	10, 20, 30, 40 and 50 eV

Figure S2. Molecular network from UHPLC-MS/MS data in the negative ion mode for *Margaritaria nobilis* leaf extract.

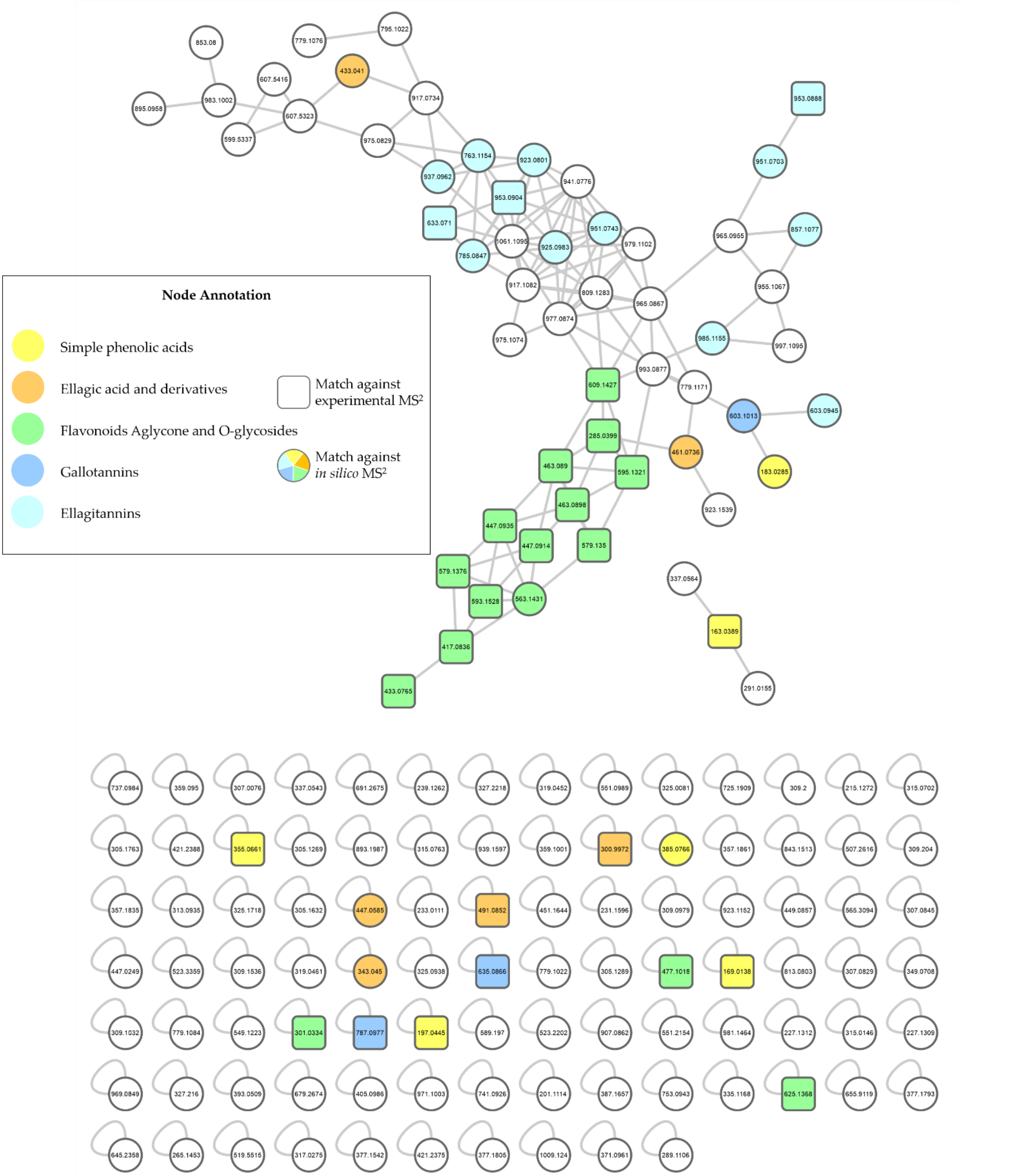

Note: Numbers inside the nodes correspond to the *m/z* accurate masses (Da) for the [M−H]⁻ for each precursor ion. Node colors represent the different groups and subgroups: Phenolic Acids derivatives (yellow: simple phenolic acids; orange: ellagic acid and derivatives), Flavonoids Aglycone and *O*-glycosides (green) and Hydrolysable Tannins (dark blue: gallotannins; light blue: ellagitannins). Roud rectangle shapes correspond to a match against an experimental MS²; colored ellipse shapes are identifications from in silico spectra matching.

Figure S3. Proposed structures for annotated metabolites in the ethanolic extract of *Margaritaria nobilis* leaves.

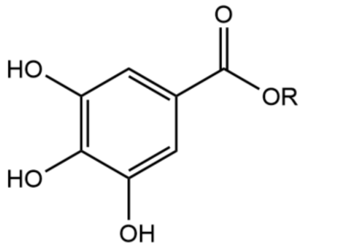

1. R = H (Gallic acid)
4. R = CH_3 (Methyl gallate)
11. R = CH_2CH_3 (Ethyl gallate)

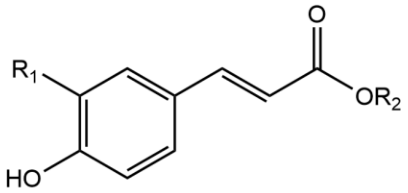

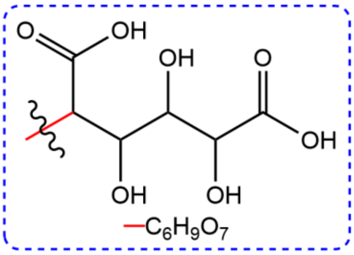

9. R_1 = R_2 = H (*p*-Coumaric acid)
2. R_1 = H | R_2 = $C_6H_9O_7$ (*O*-Coumaroylgalactaric acid)
3. R_1 = OCH_3 | R_2 = $C_6H_9O_7$ (*O*-Feruloylgalactaric acid)

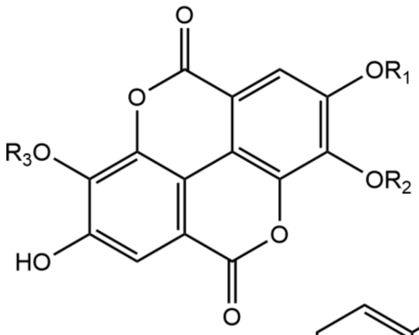

18. R_1 = R_2 = R_3 = H (Ellagic acid)
13. R_1 = R_2 = H | R_3 = Xyl (Ellagic acid *O*-xyloside)
16. R_1 = R_2 = H | R_3 = Rha (Ellagic acid *O*-rhamnoside)
27. R_1 = R_2 = CH_3 | R_3 = Glc (Di-*O*-Methyl ellagic acid *O*-glucoside)
36. R_1 = H | R_2 = CH_3 | R_3 = Rha (Methylellagic acid *O*-rhamnoside)
44. R_1 = R_2 = R_3 = CH_3 (Tri-*O*-Methylellagic acid)

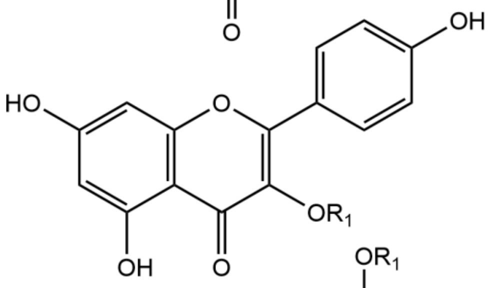

42. R_1 = H (Kaempferol)
24. R_1 = Rha-Glc (Kaempferol 3-*O*-rhamnosyl-glucoside)
26. R_1 = Xyl-Glc (Kaempferol 3-*O*-xylosyl-glucoside)
30, 33. R_1 = Glc (Kaempferol 3-*O*-glucoside Isomer I and II)
34. R_1 = Rha-Xyl (Kaempferol 3-*O*-rhamnosyl-xyloside)
35. R_1 = Xyl (Kaempferol 3-*O*-xyloside)

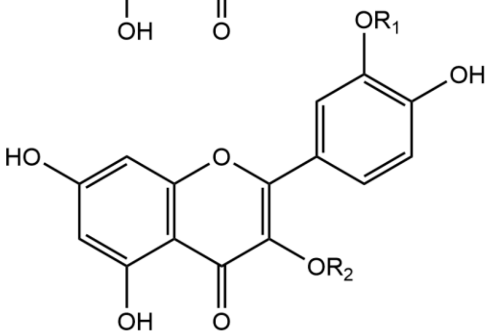

40. R_1 = R_2 = H (Quercetin)
10. R_1 = H | R_2 = Glc-Glc (Quercetin 3-*O*-glucosyl-glucoside)
14. R_1 = H | R_2 = Xyl-Glc (Quercetin 3-*O*-xylosyl-glucoside)
15. R_1 = H | R_2 = Rha-Glc (Quercetin 3-*O*-rhamnosyl-glucoside)
21, 25. R_1 = H | R_2 = Glc (Quercetin 3-*O*-glucoside Isomer I and II)
28. R_1 = H | R_2 = Rha-Xyl (Quercetin 3-*O*-rhamnosyl-xyloside)
29. R_1 = H | R_2 = Xyl (Quercetin 3-*O*-xyloside)
41. R_1 = CH_3 | R_2 = Glc (Methylquercetin 3-*O*-glucoside)

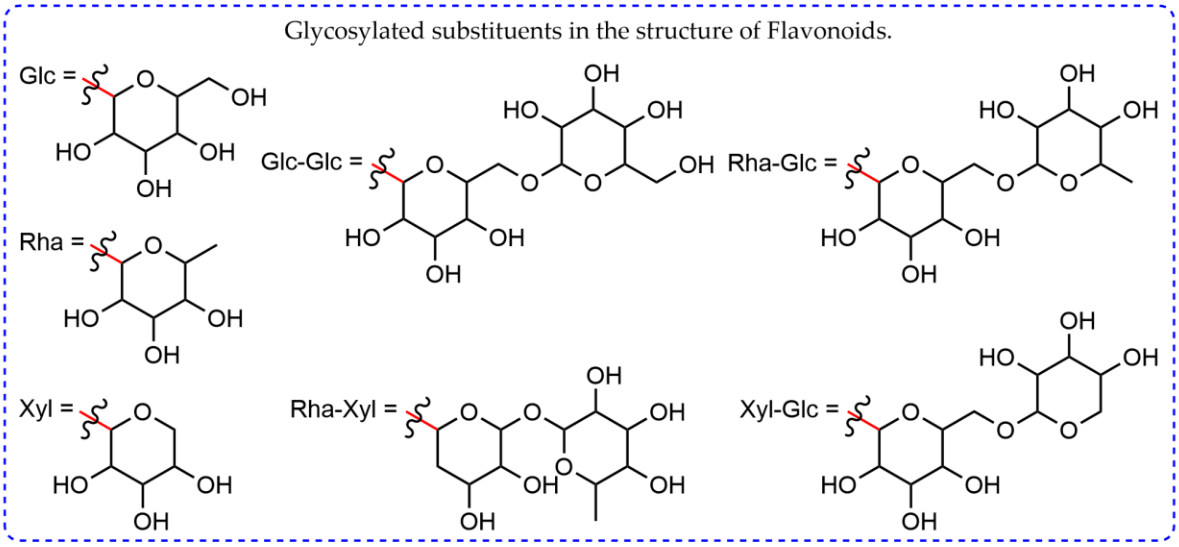

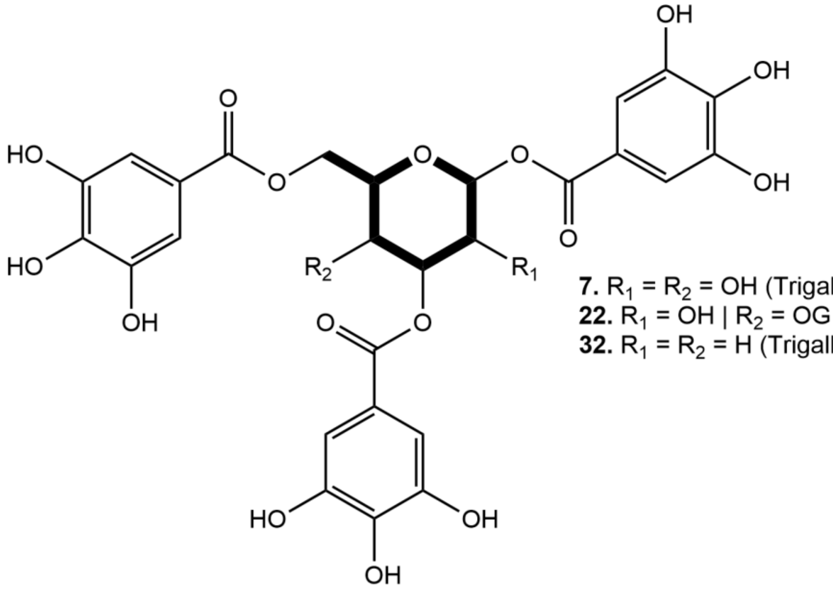

7. $R_1 = R_2$ = OH (Trigalloyl-glucose)
22. R_1 = OH | R_2 = OG (Tetragalloyl-glucose)
32. $R_1 = R_2$ = H (Trigalloyl-dideoxyglucose)

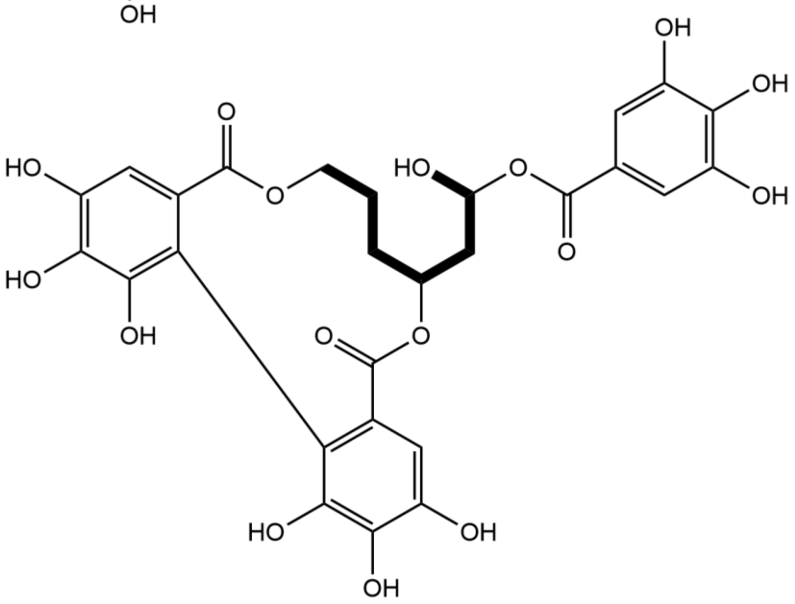

31. Galloyl-HHDP-dideoxyglucose

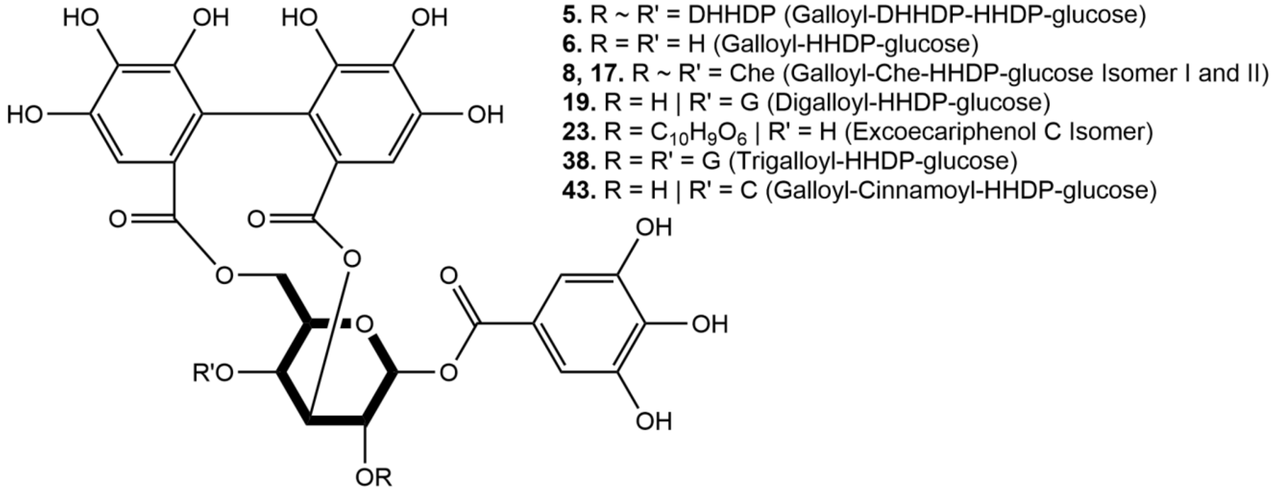

5. R ~ R' = DHHDP (Galloyl-DHHDP-HHDP-glucose)
6. R = R' = H (Galloyl-HHDP-glucose)
8, 17. R ~ R' = Che (Galloyl-Che-HHDP-glucose Isomer I and II)
19. R = H | R' = G (Digalloyl-HHDP-glucose)
23. R = $C_{10}H_9O_6$ | R' = H (Excoecariphenol C Isomer)
38. R = R' = G (Trigalloyl-HHDP-glucose)
43. R = H | R' = C (Galloyl-Cinnamoyl-HHDP-glucose)

Note: C–C bonds of glucose in bold.

12. Phyllanthusiin C Isomer
R~R' =

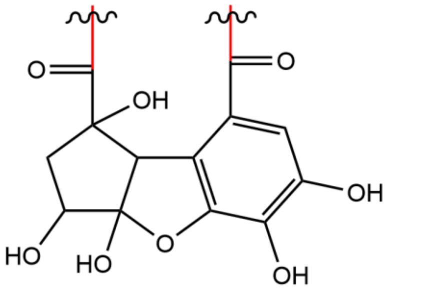

37. Phyllanthusiin A Isomer
R~R' =

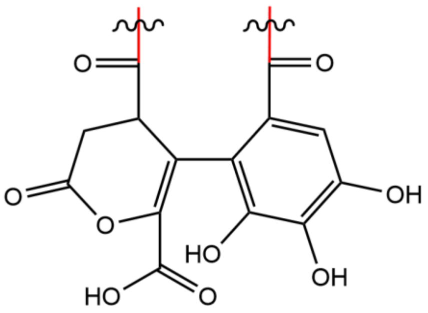

20. Methyl neochebulagate Isomer
R = H | R' =

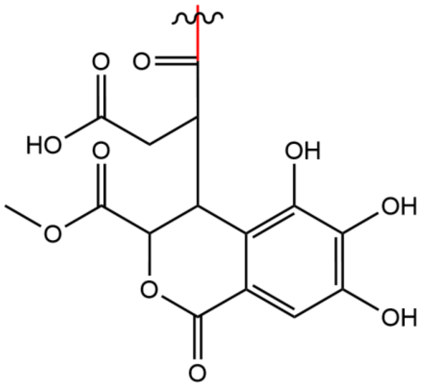

39. Phyllanthusiin U Isomer
R~R' =

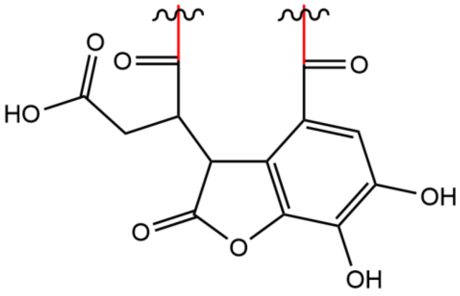

Cinnamoyl, Galloyl and its congener esters in the structure of Hydrolysable Tannins.

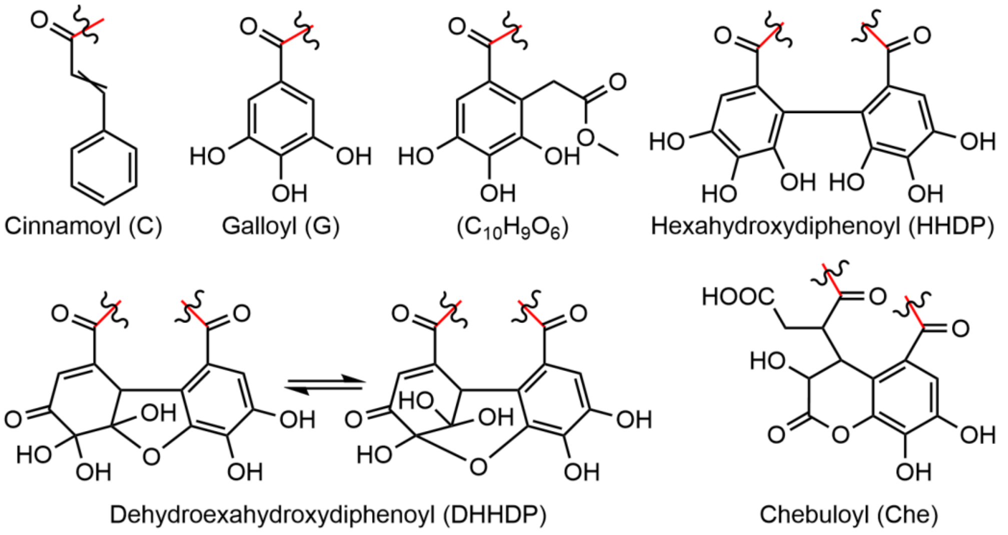

Note: Proposed structures for ions of interest using as an initial reference point the structure of compounds of the class already reported in the genus or family of *M. nobilis*, with priority given to elemental compositions that were within +/-5 ppm of the calculated values.

Figure S4. General fragmentation scheme and MS/MS spectra of *O*-glycosylated kaempferol derivatives.

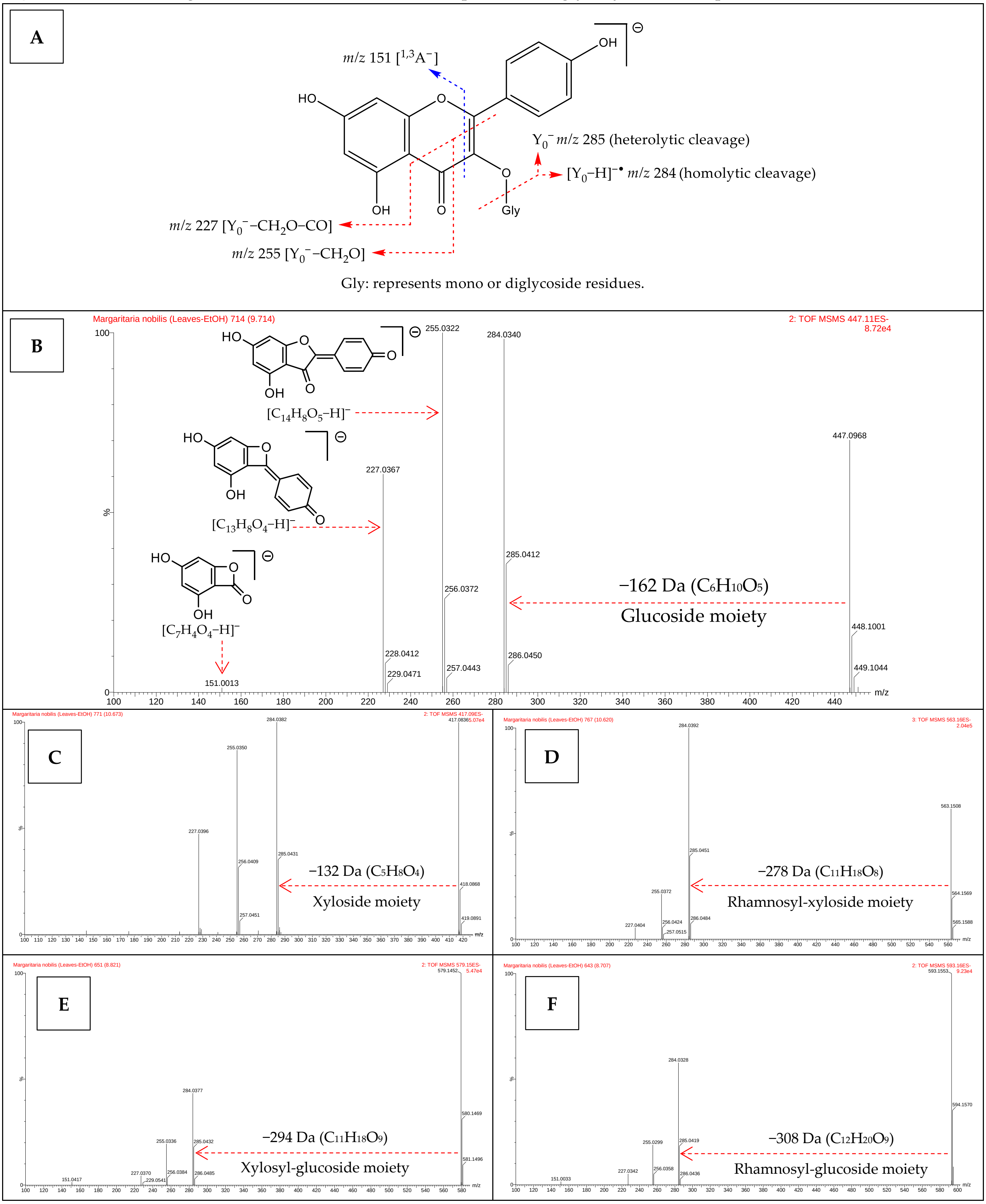

Note: (**A**) General fragmentation scheme of *O*-glycosylated kaempferol derivatives; (**B**) Kaempferol *O*-glucoside MS/MS spectrum and its main fragments; MS/MS spectra of: (**C**) Kaempferol *O*-xyloside; (**D**) Kaempferol *O*-rhamnosyl-xyloside; (**E**) Kaempferol *O*-xylosyl-glucoside and (**F**) Kaempferol *O*-rhamnosyl-glucoside.

Figure S5. *O*-glycosylated quercetin derivatives MS/MS spectra.

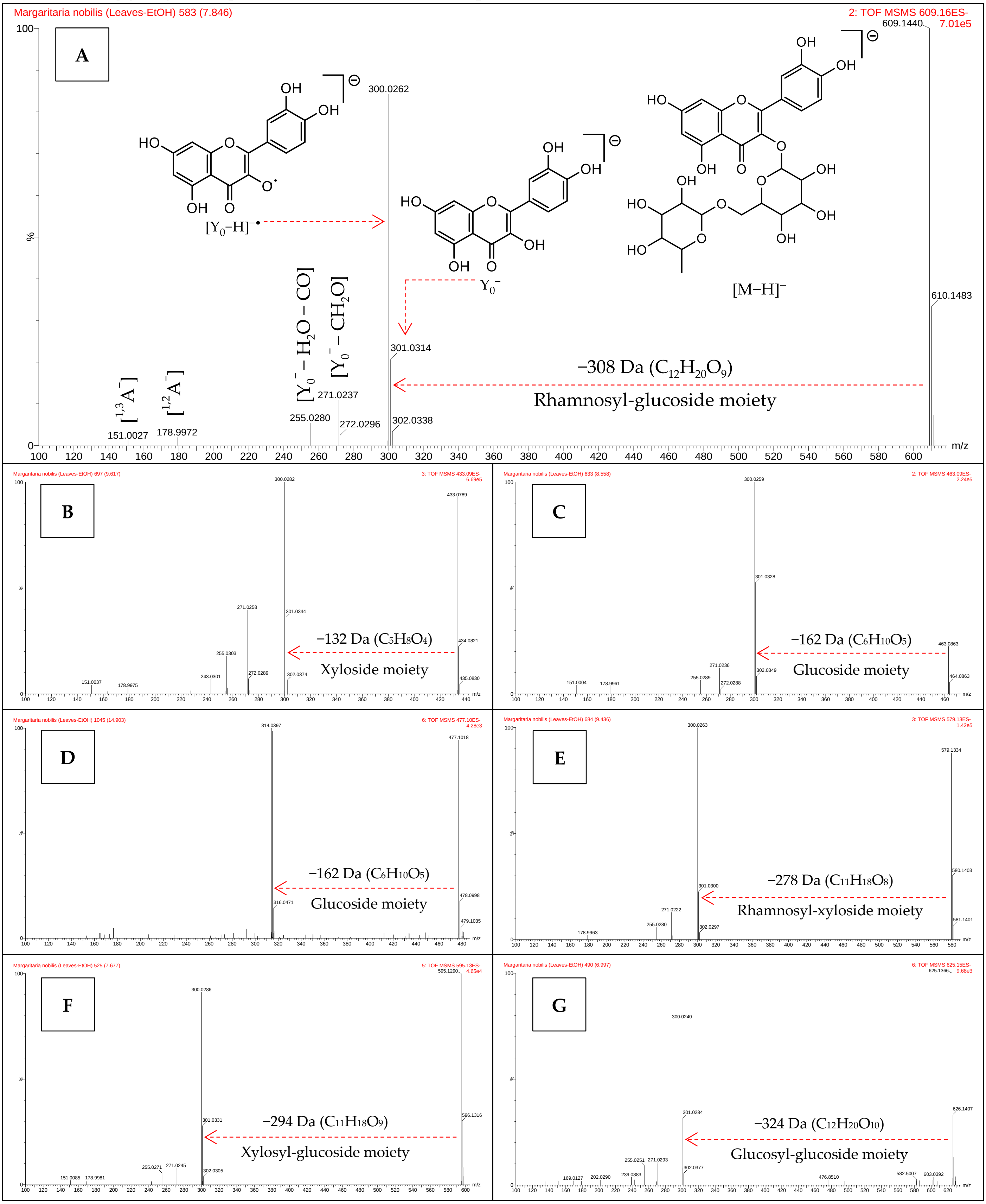

Note: (**A**) Quercetin *O*-rhamnosyl-glucoside espectra and its main fragments; MS/MS espectra of: (**B**) quercetin *O*-xyloside; (**C**) quercetin *O*-glucoside; (**D**) methylquercetin *O*-glucoside; (**E**) quercetin *O*-rhamnosyl-xyloside; (**F**) Quercetin *O*-xylosyl-glucoside and (**G**) quercetin *O*-glucosyl-glucoside.

Figure S6. MS/MS spectra of hydrolysable tannins annotated *in silico*.

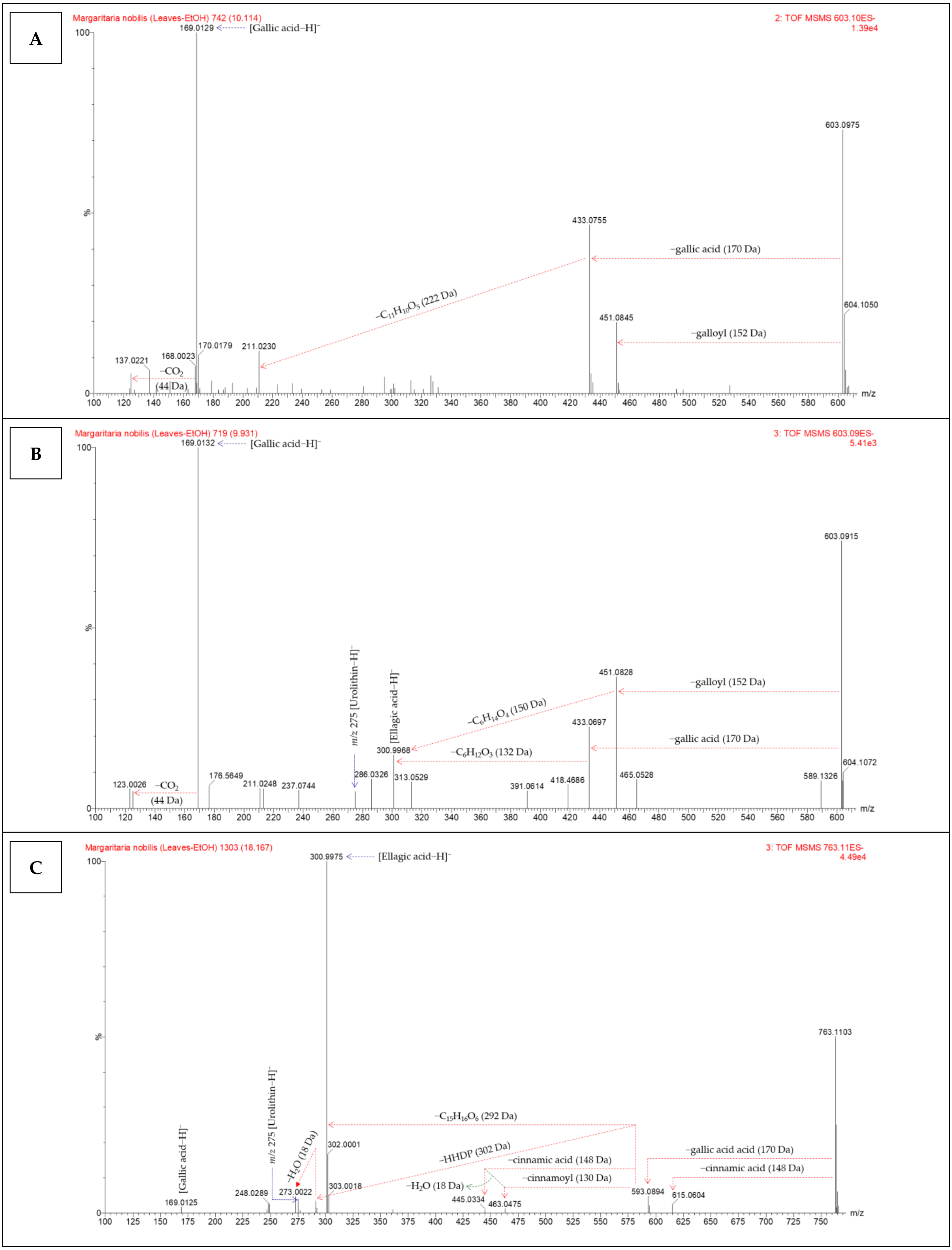

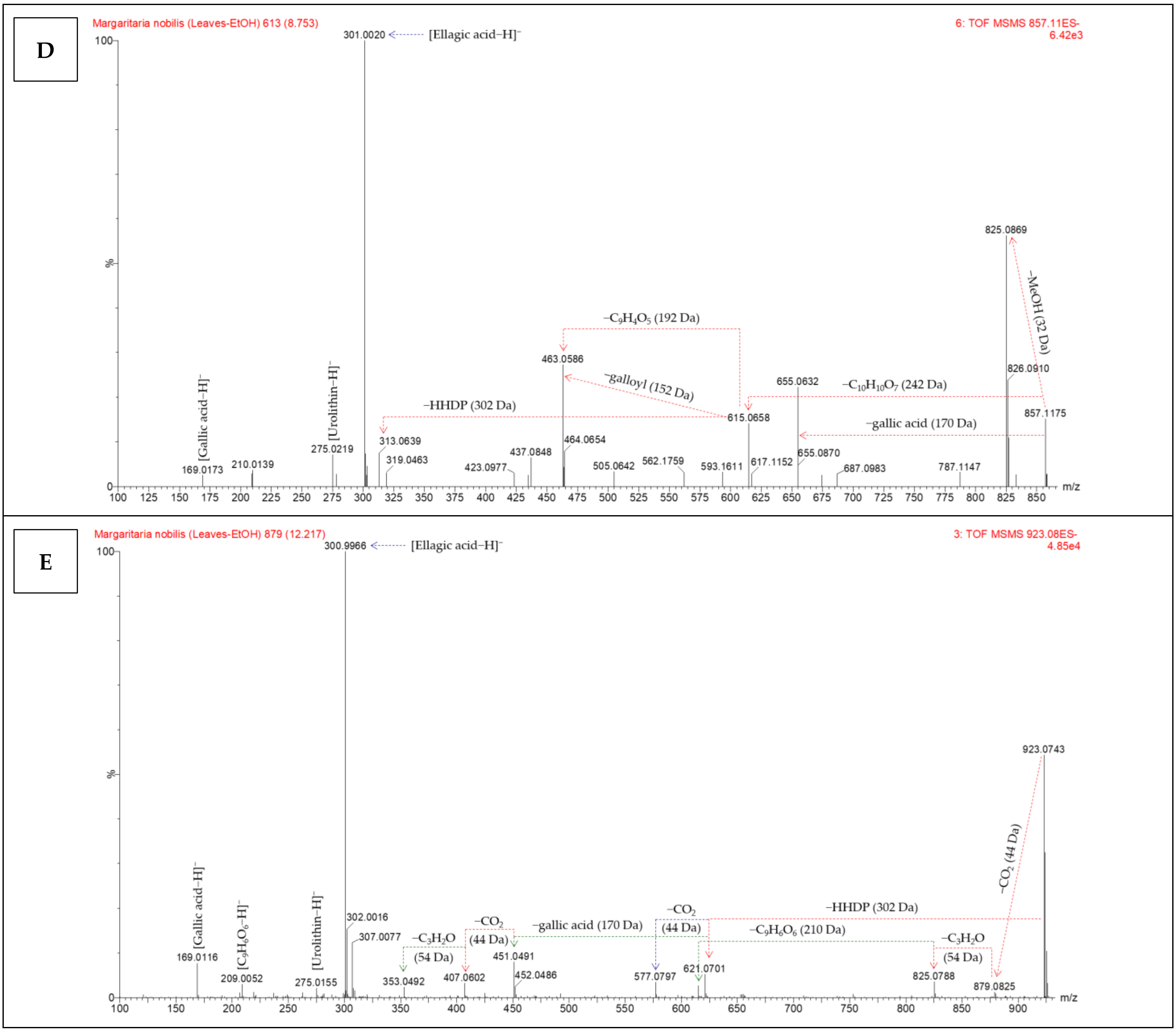

Note: (**A**) Trigalloyl-dideoxyglucose; (**B**) Galloyl-HHDP-dideoxyglucose; (**C**) Galloyl-Cinnamoyl-HHDP-glucose; (**D**) Excoecariphenol C Isomer and (**E**) Phyllanthusiin U Isomer.

Table S2. In-house database of compounds reported in the genus *Margaritaria* (Phyllanthaceae).

ID	Compound	Molecular Formula	$[M-H]^-$ Calculated	InChI	Species	Reference
**1**	4-Hydroxybenzoic acid	$C_7H_6O_3$	137.0239	InChI=1S/C7H6O3/c8-6-3-1-5(2-4-6)7(9)10/h1-4,8H,(H,9,10)	MD	[1]
**2**	3-Hydroxy-4-methoxybenzaldehyde	$C_8H_8O_3$	151.0395	InChI=1S/C8H8O3/c1-11-8-3-2-6(5-9)4-7(8)10/h2-5,10H,1H3	MD	[1]
**3**	2-Hydroxyphenylacetic acid	$C_8H_8O_3$	151.0395	InChI=1S/C8H8O3/c9-7-4-2-1-3-6(7)5-8(10)11/h1-4,9H,5H2,(H,10,11)	MD	[1]
**4**	4-Hydroxyphenylacetic acid	$C_8H_8O_3$	151.0395	InChI=1S/C8H8O3/c9-7-3-1-6(2-4-7)5-8(10)11/h1-4,9H,5H2,(H,10,11)	MD	[1]
**5**	3,4-Dihydroxybenzoic acid	$C_7H_6O_4$	153.0188	InChI=1S/C7H6O4/c8-5-2-1-4(7(10)11)3-6(5)9/h1-3,8-9H,(H,10,11)	MD	[1]
**6**	Vanillic acid	$C_8H_8O_4$	167.0344	InChI=1S/C8H8O4/c1-12-7-4-5(8(10)11)2-3-6(7)9/h2-4,9H,1H3,(H,10,11)	MD	[1]
**7**	Gallic acid	$C_7H_6O_5$	169.0137	InChI=1S/C7H6O5/c8-4-1-3(7(11)12)2-5(9)6(4)10/h1-2,8-10H,(H,11,12)	MD, MN	[1,2]
**8**	Methyl gallate	$C_8H_8O_5$	183.0293	InChI=1S/C8H8O5/c1-13-8(12)4-2-5(9)7(11)6(10)3-4/h2-3,9-11H,1H3	MN	[2]
**9**	2-Hydroxy-2-(2-hydroxyphenyl)acetic acid ethyl ester	$C_{10}H_{12}O_4$	195.0657	InChI=1S/C10H12O4/c1-2-14-10(13)9(12)7-5-3-4-6-8(7)11/h3-6,9,11-12H,2H2,1H3	MD	[1]
**10**	Vanillyl glycol	$C_{10}H_{14}O_4$	197.0814	InChI=1S/C10H14O4/c1-14-10-5-7(2-3-9(10)13)4-8(12)6-11/h2-3,5,8,11-13H,4,6H2,1H3	MD	[1]
**11**	Dodecanoic acid	$C_{12}H_{24}O_2$	199.1698	InChI=1S/C12H24O2/c1-2-3-4-5-6-7-8-9-10-11-12(13)14/h2-11H2,1H3,(H,13,14)	MD	[1]

**12**	Securinine	$C_{13}H_{15}NO_2$	216.1025	InChI=1S/C13H15NO2/c15-12-7-9-4-5-10-8-13(9,16-12)11-3-1-2-6-14(10)11/h4-5,7,10-11H,1-3,6,8H2/t10-,11-,13+/m1/s1	MD	[1]
**13**	Allosecurinin	$C_{13}H_{15}NO_2$	216.1025	InChI=1S/C13H15NO2/c15-12-7-9-4-5-10-8-13(9,16-12)11-3-1-2-6-14(10)11/h4-5,7,10-11H,1-3,6,8H2	MI	[3]
**14**	(-)-Securinine	$C_{13}H_{15}NO_2$	216.1025	InChI=1S/C13H15NO2/c15-12-7-9-4-5-10-8-13(9,16-12)11-3-1-2-6-14(10)11/h4-5,7,10-11H,1-3,6,8H2/t10-,11-,13+/m1/s1	MI	[3]
**15**	(+)-Philantidine	$C_{13}H_{15}NO_3$	232.0974	InChI=1S/C13H15NO3/c15-12-7-9-4-5-10-8-13(9,16-12)11-3-1-2-6-14(11)17-10/h4-5,7,10-11H,1-3,6,8H2	MN	[2]
**16**	(+)-15-Methoxy-14,15-dihydrophyllocrisy	$C_{14}H_{19}NO_3$	248.1287	InChI=1S/C14H19NO3/c1-17-11-6-9-7-13(16)18-14(9)8-10(11)15-5-3-2-4-12(14)15/h7,10-12H,2-6,8H2,1H3/t10?,11?,12?,14-/m0/s1	MI	[3]
**17**	Kaempferol	$C_{15}H_{10}O_6$	285.0399	InChI=1S/C15H10O6/c16-8-3-1-7(2-4-8)15-14(20)13(19)12-10(18)5-9(17)6-11(12)21-15/h1-6,16-18,20H	MN	[2]
**18**	Epicatechin	$C_{15}H_{14}O_6$	289.0712	InChI=1S/C15H14O6/c16-8-4-11(18)9-6-13(20)15(21-14(9)5-8)7-1-2-10(17)12(19)3-7/h1-5,13,15-20H,6H2/t13-,15-/m1/s1	MD	[1]
**19**	Catechin	$C_{15}H_{14}O_6$	289.0712	InChI=1S/C15H14O6/c16-8-4-11(18)9-6-13(20)15(21-14(9)5-8)7-1-2-10(17)12(19)3-7/h1-5,13,15-20H,6H2/t13-,15+/m0/s1	MD	[1]
**20**	Epigallocatechin	$C_{15}H_{14}O_7$	305.0661	InChI=1S/C15H14O7/c16-7-3-9(17)8-5-12(20)15(22-13(8)4-7)6-1-	MD	[1]

				10(18)14(21)11(19)2-6/h1-4,12,15-21H,5H2/t12-,15-/m1/s1		
**21**	Gallocatechin	$C_{15}H_{14}O_7$	305.0661	InChI=1S/C15H14O7/c16-7-3-9(17)8-5-12(20)15(22-13(8)4-7)6-1-10(18)14(21)11(19)2-6/h1-4,12,15-21H,5H2/t12-,15+/m0/s1	MD	[1]
**22**	3-Methoxy-4-hydroxybenzeneacetic acid	$C_{18}H_{18}O_8$	361.0923	InChI=1S/C18H18O8/c1-25-13-5-9(7-15(19)20)3-11(17(13)23)12-4-10(8-16(21)22)6-14(26-2)18(12)24/h3-6,23-24H,7-8H2,1-2H3,(H,19,20)(H,21,22)	MD	[1]
**23**	Betulinic acid	$C_{30}H_{48}O_3$	455.3525	InChI=1S/C30H48O3/c1-18(2)19-10-15-30(25(32)33)17-16-28(6)20(24(19)30)8-9-22-27(5)13-12-23(31)26(3,4)21(27)11-14-29(22,28)7/h19-24,31H,1,8-17H2,2-7H3,(H,32,33)/t19-,20+,21-,22+,23-,24+,27-,28+,29+,30-/m0/s1	MN	[2]
**24**	Phagovatin	$C_{28}H_{32}O_{14}$	591.1714	InChI=1S/C28H32O14/c1-10-20(31)23(34)26(37)28(40-10)39-9-17-21(32)24(35)25(36)27(42-17)19-15(38-2)8-16-18(22(19)33)13(30)7-14(41-16)11-3-5-12(29)6-4-11/h3-8,10,17,20-21,23-29,31-37H,9H2,1-2H3/t10?,17?,20-,21+,23-,24-,25?,26?,27-,28+/m0/s1	MD	[4]
**25**	Hydroxygenkwanin-8-C-[α-rhamnopyranosyl-(1→6)] β-glucopyranoside	$C_{28}H_{32}O_{15}$	607.1663	N/A	MD	[4]
**26**	Corilagin	$C_{27}H_{22}O_{18}$	633.0728	InChI=1S/C27H22O18/c28-9-1-6(2-10(29)16(9)32)24(39)45-27-22(38)23-	MN	[2]

				19(35)13(43-27)5-42-25(40)7-3-11(30)17(33)20(36)14(7)15-8(26(41)44-23)4-12(31)18(34)21(15)37/h1-4,13,19,22-23,27-38H,5H2/t13-,19-,22-,23+,27+/m1/s1		
**27**	Kaempferol-3-*O*-α-rhamnopyranosyl-(1→2)-β-glucopyranoside-7-*O*-α-rhamnopyranoside	$C_{33}H_{40}O_{19}$	739.2086	InChI=1S/C33H40O19/c1-10-19(37)23(41)26(44)31(46-10)48-14-7-15(36)18-16(8-14)49-28(12-3-5-13(35)6-4-12)29(22(18)40)51-33-30(25(43)21(39)17(9-34)50-33)52-32-27(45)24(42)20(38)11(2)47-32/h3-8,10-11,17,19-21,23-27,30-39,41-45H,9H2,1-2H3/t10-,11-,17+,19-,20-,21+,23+,24+,25-,26+,27+,30+,31-,32-,33-/m0/s1	MD	[4]
**28**	Kaempferol-3-*O*-α-rhamnopyranosyl-(1→2)-[α-rhamnopyranosyl-(1→6)]-β-glucopyranoside-7-O-α-rhamnopyranoside	$C_{39}H_{50}O_{23}$	885.2665	InChI=1S/C39H50O23/c1-11-21(42)25(46)29(50)37(55-11)58-16-8-17(41)20-18(9-16)59-34(14-4-6-15(40)7-5-14)35(24(20)45)62-39-31(52)27(48)23(44)19(60-39)10-54-36-32(53)28(49)33(13(3)57-36)61-38-30(51)26(47)22(43)12(2)56-38/h4-9,11-13,19,21-23,25-33,36-44,46-53H,10H2,1-3H3/t11?,12?,13?,19?,21-,22-,23-,25-,26-,27?,28+,29?,30?,31?,32?,33-,36+,37-,38-,39-/m0/s1	MD	[4]

Note: (The data were reported in the literature 1990-2015 - Source: Scopus, Web of Science, Science Direct, Scielo, PubMed and Google Scholar). N/A: Not Avaliable; MN: *Margaritaria nobilis*; MD: *Margaritaria discoidea*; MI: *Margaritaria indica*.

References

1. Johnson-Ajinwo, O.R.; Richardson, A.; Li, W.-W. Cytotoxic Effects of Stem Bark Extracts and Pure Compounds from *Margaritaria discoidea* on Human Ovarian Cancer Cell Lines. *Phytomedicine* **2015**, *22*, 1–4, doi:10.1016/j.phymed.2014.09.008.
2. Moraes, L.S.; Donza, M.R.H.; Rodrigues, A.P.D.; Silva, B.J.M.; Brasil, D.S.B.; Zoghbi, M.D.G.B.; Andrade, E.H.A.; Guilhon, G.M.S.P.; Silva, E.O.; Schmidt, T.J. Leishmanicidal Activity of (+)-Phyllanthidine and the Phytochemical Profile of *Margaritaria nobilis* (Phyllanthaceae). *Molecules* **2015**, *20*, 22157–22169, doi:10.3390/molecules201219829.
3. Arbain, D.; Byrne, L.; Cannon, J.; Engelhardt, L.; White, A. The Alkaloids of *Margaritaria indica* (Euphorbiaceae). The Crystal Structure and Absolute Configuration of the Hydrobromide of (+)-15α-Methoxy-14,15-Dihydrophyllochrysine. *Australian Journal of Chemistry* **1990**, *43*, 439, doi:10.1071/CH9900439.
4. Ekuadzi, E.; Dickson, R.; Fleischer, T.; Annan, K.; Pistorius, D.; Oberer, L.; Gibbons, S. Flavonoid Glycosides from the Stem Bark of *Margaritaria discoidea* Demonstrate Antibacterial and Free Radical Scavenging Activities. *Phytotherapy Research* **2014**, *28*, 784–787, doi:10.1002/ptr.5053.
